# Progressive Induction of Type 2 Diabetes: Effects of a Reality–Like Fructose Enriched Diet in Young Wistar Rats

**DOI:** 10.1371/journal.pone.0146821

**Published:** 2016-01-22

**Authors:** Julie Dupas, Christelle Goanvec, Annie Feray, Anthony Guernec, Charlène Alain, François Guerrero, Jacques Mansourati

**Affiliations:** 1 Optimisation des Régulations Physiologiques, Université de Bretagne Occidentale, Brest, France; 2 UFR Sciences et Techniques, Université de Bretagne Occidentale, Brest, France; 3 UFR Sport et Education Physique, Université de Bretagne Occidentale, Brest, France; 4 Département de Cardiologie, Centre Hospitalo-Universitaire de Brest, Brest, France; University of Catanzaro Magna Graecia, ITALY

## Abstract

**Purpose:**

The aim of this study was to characterize short and medium-lasting effects of fructose supplementation on young Wistar rats. The diet was similar to actual human consumption.

**Methods:**

Three week old male rats were randomly divided into 2 groups: control (C; n = 16), fructose fed (FF; n = 16) with a fructose enriched drink for 6 or 12 weeks. Bodyweight, fasting glycemia and systolic blood pressure were monitored. Glucose tolerance was evaluated using an oral glucose tolerance test. Insulinemia was measured concomitantly and enable us to calculate insulin resistance markers (HOMA-IR, Insulin Sensitivity Index for glycemia: ISI-gly). Blood chemistry analyses were performed.

**Results:**

After six weeks of fructose supplementation, rats were not overweight but presented increased fasting glycemia, reduced glucose tolerance, and lower insulin sensitivity compared to control group. Systolic blood pressure and heart weight were also increased without any change in renal function (theoretical creatinine clearance). After twelve weeks of fructose supplementation, FF rats had increased bodyweight and presented insulin resistance (higher HOMA-IR, lower ISI-gly). Rats also presented higher heart volume and lower ASAT/ALAT ratio (presumed liver lesion). Surprisingly, the Total Cholesterol/Triglycerides ratio was increased only after six weeks of fructose supplementation, predicting a higher LDL presence and thus a higher risk of developing cardiovascular disease. This risk was no longer present after twelve weeks of a fructose enriched diet.

**Conclusion:**

On young Wistar rats, six weeks of fructose supplementation is sufficient to induce signs of metabolic syndrome. After twelve weeks of fructose enriched diet, rats are insulin resistant. This model enabled us to study longitudinally the early development of type 2 diabetes.

## Introduction

Type 2 diabetes (T2D) is a metabolic disease defined by a fasting hyperglycemia frequently related to the progressive development of resistance towards insulin. According to the World Health Organization (WHO), the number of diabetic patients increased from 30 million in 1985 to 366 million in 2011 [1]. This number is expected to rise to 552 million in 2030. Therefore diabetes is considered epidemic [2]. The rapid increase in diabetes prevalence is strongly related to changes in lifestyle including reduced physical activity and dietary changes [1]. The development of T2D as well as other metabolic syndrome parameters (hypertension, dyslipidemia and obesity) are strongly related to the high consumption of sugar [3] and more specifically fructose consumption [4]. Fructose is commonly used as a sweetener for pastries and more often sweetened beverages like soft drinks.

In rodents, mainly Sprague Dawley rats, a diet high in fructose is already known to induce symptoms of metabolic syndrome and even T2D [5–9]. However, Sprague-Dawley rat are more sensitive towards T2D than Wistar rats [9,10]. In the human population, as in Wistar rats, sensitivity towards T2D varies greatly from one individual to another. These studies mainly used a diet which fructose content was composed of 60–66% fructose which is not representative of human consumption [4].

Little is known about the effects of fructose on the development of T2D in Wistar rats. Indeed, published studies differ greatly in terms of results. The diversity of study designs can be a possible explanation. Amongst the varying factors, age at the beginning of the study, duration of the study, quantity of fructose and administration methods can be mentioned. Thus, the aims of this study were first to develop a new model of Wistar rats representative of human fructose consumption and then to evaluate the impact of such consumption on the development of type 2 diabetes. As Tappy and Lê, (2010) [4] demonstrated, humans consume fructose mainly with sweet drinks, and began their high fructose consumption at very young age reaching the highest during teenage years and early adult life (19–22 years).

## Materials and Methods

### Animals

Experiments followed the French "Ministère de l'Éducation Nationale, de l'Enseignement Supérieur et de la Recherche" guidelines. The French "Ministère de l'Éducation Nationale, de l'Enseignement Supérieur et de la Recherche" approved this experiment (authorization n°2269). Rats were euthanized with Ketamine (Ketamine 100, Virbac, 80mg.kg-1)/Xylazine (Rompun 2%, Bayer, 12mg.kg-1) injected intramuscularly (into the left back leg), the detailed procedure is described below.

32 Male Wistar rats (Janvier labs), 3 weeks old (weight under 49g, Janvier labs, Le Genest Saint Ile, France), were housed individually in a light (12h:12h light/dark cycle) and temperature (21°C ± 1°C) controlled animal facility. Rats were all fed with a standard chow (Kliba Nafag, M/R Maintenance). Randomly chosen rats had either tap water (C; n = 16) or a fructose enriched drink (20% w/v from age 3 weeks to 9 weeks and 25% w/v from age 10 weeks to 15 weeks) (FF; n = 16). Fructose enriched drinks were changed every couple of days; water bottles were sterilized every week.

Rats were weighed weekly. To study the progressive effects of fructose supplementation on young Wistar rats, after the first six weeks of a fructose enriched diet (age: 9 weeks) half of the rats in each group (n = 8) were euthanized, the other half remained in the study for another six weeks before being euthanized (age: 15 weeks). Rats were euthanized with Ketamine (Ketamine 100, Virbac, 80mg.kg^-1^)/Xylazine (Rompun 2%, Bayer, 12mg.kg^-1^) injected intramuscularly (into the left back leg). Blood was collected intraventricularly, plasma was then obtained after 15min at 1000g centrifugation and immediately frozen in liquid nitrogen and stored at -80°C until further analysis. The hearts were collected, and residual blood was removed and then weighed to calculate the heart volume. The gastrocnemius and left ventricle were collected for antioxidant enzyme measurements and immediately frozen in liquid nitrogen and then stored at -80°C before measurements.

The heart volume (% body weight) was calculated as followed:

$$\text{heart volume }\left(\text{\% bw}\right)=\frac{\text{heart weight }\left(\text{g}\right)}{\text{body weight }\left(\text{g}\right)}\times 100$$

Systolic blood pressure was measured at 8 weeks, using a tail cuff blood pressure system (Model 29 pulse amplifier with tail cuff sensor and adapted rodent restrainer, iitc incorporated). Rats were acclimated to the blood pressure system for 4 days before measurement.

### Glucose tolerance and insulin resistance measurements

After 15h fasting, glucose was measured in blood collected by a single prick onto the mandibular veins (allowing only one drop to come off) using a glucometer (Accu-Chek Performa, Roche, Meylan, France)[11].

For oral glucose tolerance test (OGTT), tips of the tails were anesthetized using a local anaesthetic (Anesderm 5% Gé, Lidocaïne (2.5%), Prilocaïne (2.5%), Pierre Fabre Dermatologie) and then cut to allow the blood to flow. The first drop was used to measure fasting glycemia (0min). A high dose of glucose (1g/kg) was then given to ingest using 0.5g/mL glucose syrup. Glycemia was then monitored for 2 hours with measurements taken at 15min, 30min, 45min, 60min, 90min and 120min after the glucose ingestion. During OGTT, blood samples (250–300μL) were collected into Lithium-Heparin tube (Microvette, Sarstedt) from the tail each time glycemia was evaluated (0, 15, 30, 45, 60, 90 and 120min). OGTT were performed twice: at age 9 and 13 weeks.

Plasma from the OGTT blood sample was obtained after 5min centrifugation at 2000g. Plasma was then frozen and stored at -80°C before further analysis. Insulin concentration was evaluated on those plasma samples using ELISA methods (Rat Insulin Elisa, ALPCO, Eurobio, Courtaboeuf, France).

From OGTT and insulin concentration data, two insulin resistance and sensitivity indicators were determined: the Homeostatic Model Assessment for Insulin Resistance (HOMA-IR) and then the Insulin Sensitivity Indices for glycemia (ISI-gly). Matsuda *et al*. have shown that both HOMA-IR and ISI-Gly correlated with euglycemic insulin clamp, even though the correlation was better for ISI-Gly. HOMA-IR was calculated using a HOMA-IR calculator software [12], software available at https://www.dtu.ox.ac.uk/homacalculator, Oxford University) and using OGTT data, i.e. fasting insulinemia and fasting glycemia were taken at t = 0min (before the ingestion of a high dose of glucose (1g/kg bw)). ISI-gly was calculated as followed [13]:

$$\text{ISI}-\text{gly}=\;\frac{2}{\left[\left(\text{AUC glycemia }\times \text{AUC insulin}\right)+1\right]}$$

### Blood biochemistry: metabolic syndrome markers

Blood chemistry measures were done on a Koné lab 20 (Thermo Scientific) using an adapted kit for: Aspartate aminotransferase activity (ASAT)(Biomérieux), Alanine aminotransferase activity (ALAT) (Biomerieux), Creatinine (Jaffé method, Fisher Brahms), Albumin (Bromocresol green method, Biomérieux), Non-esterified fatty acid (NEFA) (Wako), Triglycerides (PAP methods, Biomérieux), total Cholesterol (Cholesterol RTU, Biomérieux). The ASAT/ALAT ratio was then calculated.

Different molar ratios were calculated as a part of the lipids levels evaluation: NEFA to cholesterol ratio [14], NEFA to Albumin ratio[15], and Cholesterol to triglycerides ratio[16].

Theoretical creatinine clearance was calculated using the Cockcroft and Gault formula [17], that has been already used in rat model [18].

$$\text{Theoretical creatinine clearance }\left(\text{ml}/\text{min}\right)=\frac{\left(140-\text{age }\left(\text{years}\right)\right)\text{ * body weight }\left(\text{kg}\right)}{\left(\text{creatinine }\left(\text{mg}/\text{dL}\right)\text{ * }72\right)}$$

### Measurements of antioxidant capacity

300mg of either the left gastrocnemius or left ventricle (LV) were homogenised in a 4°C Tris-EDTA buffer (75mM/5mM) with an ultrathurax. The homogenate was then centrifuged for 10min at 100g, 4°C. The supernatant was then centrifuged for 10min at 12000g, 4°C. The supernatant was kept at -80°C until analysis. Proteins levels were measured using the BCA method (Interchim Uptima Protein Quantification kit). An automated plate reader was used for the analysis (SAFAS, Monaco).

#### Superoxide dismutase (SOD)

SOD activity was measured indirectly using the method that inhibits the adrenaline to adenochrome reaction [19] with the xanthine/hypoxanthine reaction as a superoxide anion producer (adapted from [20]). SOD activity was measured at 480nm (Evolution 201, Thermo-Scientific) One SOD activity unit (USOD) is defined as the quantity of enzyme needed to inhibit 50% of the adenochrome production in the absence of tissue extract. SOD activity is expressed in USOD/mg protein.

#### Catalase (CAT)

Measurements of the CAT activity were done using the Cat capacity to degrade hydrogen peroxide (H2O2) in water and oxygen (H2O +O2) [21].The kinetic of H2O2 disappearance is measured using a spectrophotometer at 240nm (Evolution 201, Thermo-Scientific). CAT activity is expressed in nmol H2O2 transformed/min/mg protein.

#### Glutathion Peroxidase (GPx)

GPx activity levels are measure indirectly, using the GPx capability to reduce, in presence of reduced Glutathion (GSH), a hyperoxide to alcohol and water. This capability, when coupled with glutathione reductase reaction, enabled us to follow GPx activity by measuring the kinetic rate of NADPH oxidation to NADP+ at 340nm (Evolution 201, Thermo-Scientific) at 340nm (adapted from[22]). GPx activity is expressed in nmol NADPH oxidized/min/mg protein.

### Statistics

All results are expressed as mean ± standard error of mean (SEM). All statistics were performed using Statistica v. 10 software (StatSoft, France). Normality of population was tested using the Shapiro-Wilk test. Adapted tests were then performed (Student t test, Mann and Whitney U test, ANOVA for repeated measures). ANOVA were followed by a post hoc test (HSD).

### Supporting information

Raw data are available as supplementary material (S1 Fig, S1–S7 Tables).

## Results

### Bodyweight, fasting insulin, theoretical creatinine clearance, plasma ASAT/ALAT ratio, albumin and lipid level

Table 1 summarizes the results at age 9 and 15 weeks. At the age of 9 weeks (i.e. after 6 weeks of fructose supplementation), FF and C had the same bodyweight (bw). However, FF had a higher fasting glycemia (93.14±1.98 vs. 81.50±1.31mg/dL, p<0.001) and a higher Total Cholesterol/Triglycerides ratio (2.53±0.28 vs. 1.59±0.19, p<0.05). No other statistical difference has been found upon the measured factors (ASAT/ALAT, theoretical creatinine clearance, NEFA, total cholesterol…).

**Table 1:** Effects of fructose supplementation on bodyweight, fasting glycemia, theoretical creatinine clearance and plasma ASAT, ALAT, albumin and lipid levels.

	*9 weeks*		*15 weeks*	
	FF	C	FF	C
*Bodyweight (g)*	372.3±7.1 *n = 16*	326.8±7.3 *n = 16*	495.2±25.3 *n = 8*	408.5±14.1 ** *n = 8*
*Fasting Glycemia (g)*	93.14±1.98 *n = 8*	81.50±1.31 *** *n = 8*	98.28±3.21 *n = 7*	88.14±3.26 * *n = 7*
*ASAT/ALAT ratio*	2.19±0.08 *n = 8*	2.22±0.24 *n = 7*	2.72±0.35 *n = 8*	4.11±0.47 * *n = 7*
*Theoretical creatinine clearance (ml/min)*	1.31±0.19 *n = 8*	1.25±0.05 *n = 7*	1.59±0.07 *n = 7*	1.21±0.03 *** *n = 7*
*NEFA (μmol/L)*	424.8±11.8 *n = 8*	408.9±51.8 *n = 8*	464.83±18.4 *n = 7*	382.8±32.5 *p = 0*.*054 n = 8*
*Total cholesterol (mg/L)*	763.5±42.9 *n = 8*	771.9±26.6 *n = 8*	713.9±54.1 *n = 7*	629.2±35.8 *n = 8*
*Triglycerides (mg/L)*	1153.2±191.3 *n = 8*	731.2±81.1 *n = 8*	753.6±87.1 *n = 7*	700.9±66.9 *n = 7*
*NEFA/Total Cholesterol (molar ratio)*	0.22±0.01 *n = 8*	0.21±0.03 *n = 8*	0.27±0.02 *n = 7*	0.22±0.02 *n = 7*
*NEFA/Albumin (molar ratio)*	0.73±0.02 *n = 8*	0.66±0.08 *n = 7*	0.87±0.04 *n = 7*	0.78±0.05 *n = 7*
*Total cholesterol/Triglycerides (molar ratio)*	2.53±0.28 *n = 7*	1.59±0.19 * n = 8	1.94±0.396 *n = 7*	1.89±0.24 *n = 7*

FF: fructose supplemented; C: control. Statistical values:

* p<0.05,

** p<0.01,

***p<0.001.

At age 15 weeks (i.e. after 12 weeks of fructose supplementation), FF had a higher bodyweight than C (495.2±25.3 vs. 408.5±14.1 g, p<0.01). Fructose supplementation also increases fasting glycemia (98.28±3.21 vs. 88.14±2.26 mg/dL, p<0.05) and theoretical creatinine clearance (1.59±0.07 vs. 1.21±0.03 mL/min, p<0.001). On the other hand, fructose supplementation lowered ASAT/ALAT ratio (2.72±0.35 vs. 4.11±0.47, p<0.05). It can be noticed that the ASAT/ALAT ratio is mainly modified by ALAT levels increasing, as ASAT levels remained stable. No statistical difference has been identified at this age, although NEFA had tendency (p = 0.054) to be increased under fructose supplementation. Neither total cholesterol, nor NEFA/Total cholesterol ratio, nor NEFA/Albumin ratio were modified in our study.

### Oral glucose tolerance test and corresponding insulin levels

**Fig 1:**
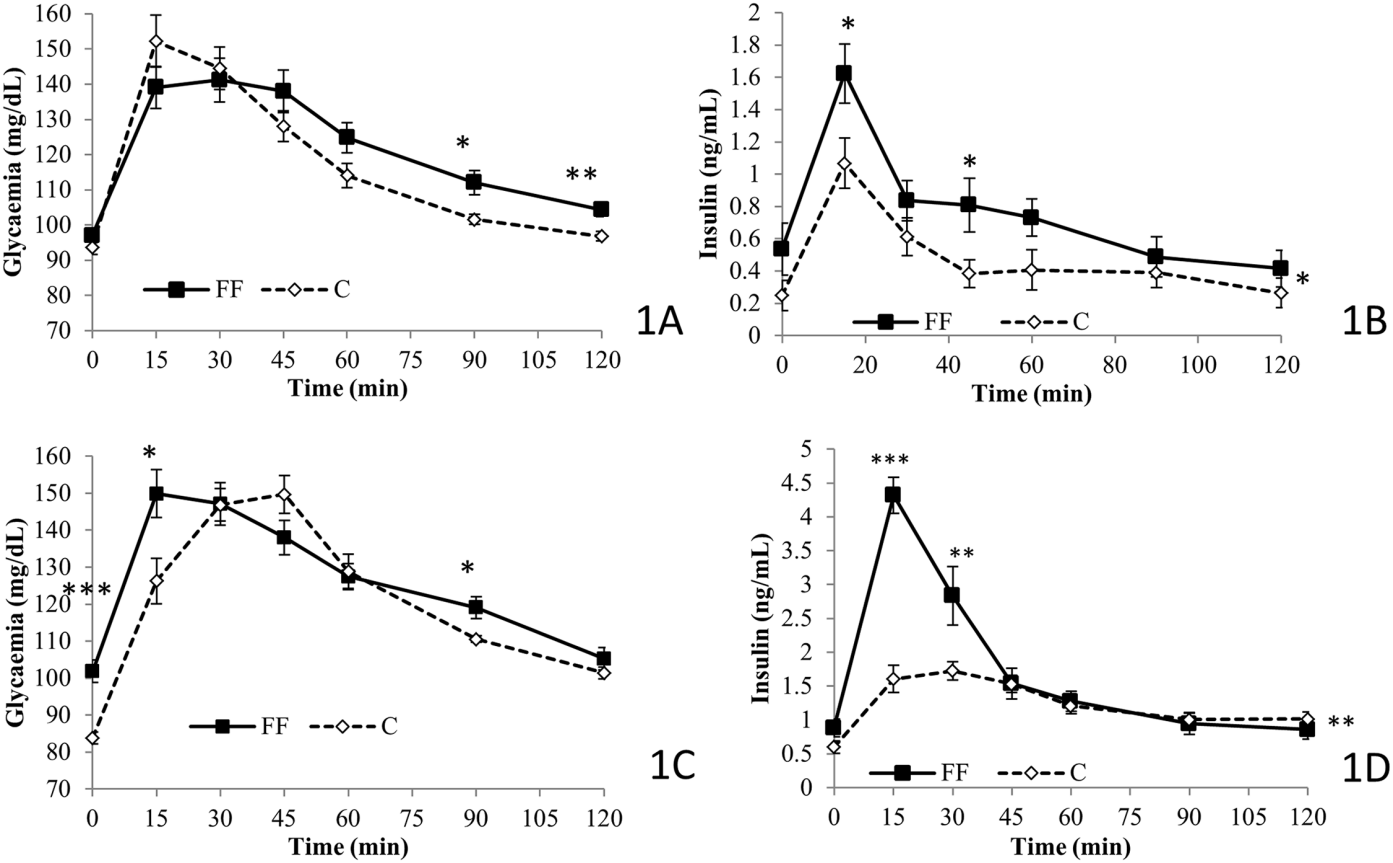
Effects of fructose supplementation on oral glucose tolerance test and its corresponding insulin level at age 9 and 13 weeks. (1 A and B) Glycemia (mg/dL) is represented as a function of the time (min); at age 9 weeks, *n = 8* for both C and FF, at age 13 weeks *n = 7* for C rats and *n = 8* for FF rats. (1 C and D) Insulin levels (ng/mL) are represented as a function of the time (min); at age 9 weeks, *n = 6* for both C and FF, at age 13 weeks *n = 7* for C rats and *n = 7* for FF rats. FF: fructose supplemented; C: control. Statistical values: *<0.05, **<0.01, ***<0.001.

OGTT was performed twice: at age 9 and 13 weeks (Fig 1A and 1B). Insulin levels were measured concomitantly (Fig 1C and 1D). At age 9 weeks, OGTT results showed that FF rats had a higher glycemia than C rats at both 90 and 120 min after the ingestion of a high dose of glucose (90min: FF 112.1±3.4 vs. C 101.6±1.4 mg/dL, p<0.05; 120min: FF 104.4±2.0 vs. C 96.8±1.4 mg/dL, p<0.01)(Fig 1A). Concomitantly insulin levels were higher at 15 and 45min after the ingestion of glucose (15min: 1.62±0.18 vs. 1.07±0.15 ng/mL; 45min: 0.81±0.17 vs. 0.38±0.08 ng/mL, both p<0.05) (Fig 1C). At age 13 weeks, OGTT results (Fig 1B) showed that compared to the control diet, fructose supplementation increased glycemia at both 15 and 90min after the ingestion of a high dose of glucose (15min: 149.9±6.5 vs. 126.3±6.1 mg/dL; 90min: 119.1±2.9 vs. 110.6±0.8 mg/dL, both p<0.05). Simultaneously, 15 and 30min after the ingestion of high dose of glucose, insulin levels were higher for the FF compared to the C (15min: 4.32±0.26 vs. 1.60±0.20 ng/mL, p<0.001; 30min: 2.84±0.43 vs. 1.72±0.13 ng/mL, p<0.01).

### Insulin resistance indicator: HOMA-IR and ISI-gly

**Fig 2:**
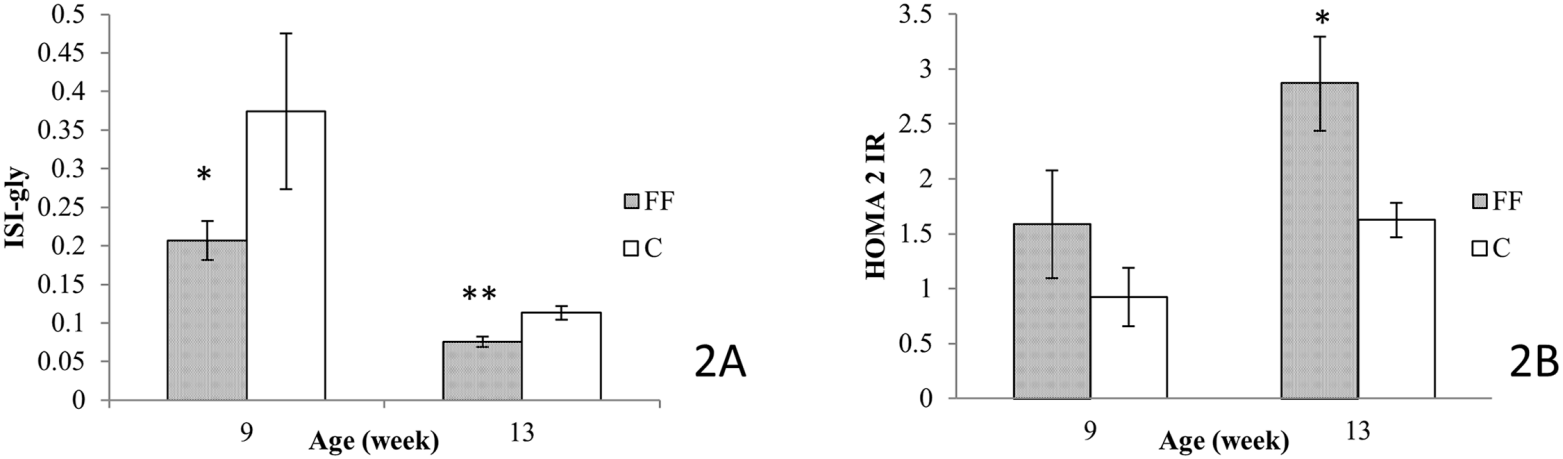
Effects of fructose supplementation on Insulin Sensitivity Indices for glycemia (ISI-gly) and Homeostasis Model Assessment for Insulin Resistance (HOMA-IR). (2A) ISI-gly was calculated at both 9 and 13 weeks. (2B) HOMA-IR was calculated at both 9 and 13 weeks. FF: fructose supplemented; C: control. For both indicator: at age 9 weeks *n = 6* for both C and FF, at age 13 weeks *n = 7* for C rats and *n = 7* for FF rats. Statistical values: *<0.05, **<0.01, ***<0.001.

HOMA-IR (Fig 2A) and ISI-gly (Fig 2B) were calculated from OGTT data. At age 9 weeks, compared to the control diet, fructose supplementation has no statistically relevant effect on HOMA-IR (p = 0.07), however this supplementation reduced ISI-gly values (FF: 0.21±0.02 vs. C: 0.37±0.10, p<0.05). Interestingly, at age 13 weeks, fructose supplementation had statistically significant effects on both HOMA-IR and ISI-gly. Indeed, FF rats had a higher HOMA-IR value than C rats (2.87±0.43 vs. 1.62±0.15, p<0.05), and had lower values of ISI-gly (0.076±0.007 vs. 0.113±0.009, p<0.01).

### Systolic blood pressure and heart volume

**Fig 3:**
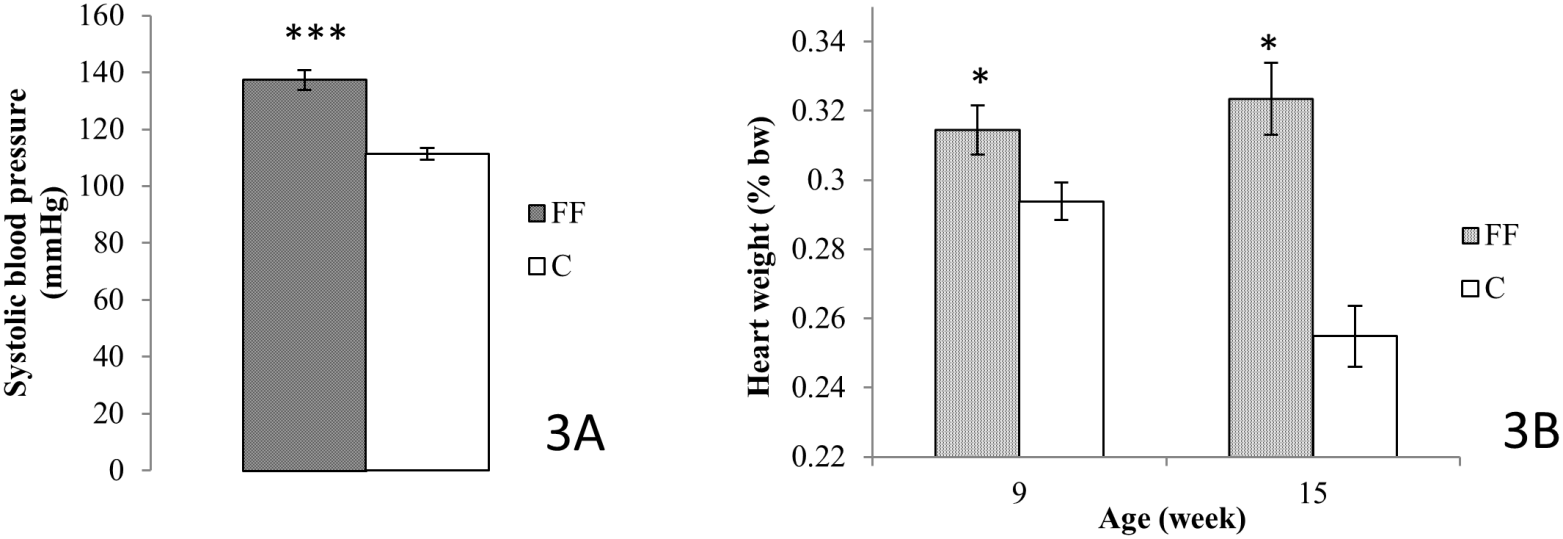
Effects of fructose supplementation on systolic blood pressure and heart volume. (3A) Systolic blood pressure was measure at age 8 weeks (*n = 8* for both FF and C). (3B) Heart volume was measured at age 9 and 15 weeks; at age 9 weeks *n = 8* for both C and FF rats, at age 15 weeks *n = 7* for both groups. FF: fructose supplemented; C: control. Statistical values: *<0.05, **<0.01, ***<0.001.

Systolic blood pressure (Fig 3A) was only measured at 8 weeks of age. In FF rats systolic blood pressure was significantly increased (FF: 137.3±3.4 vs. C: 111.2±2.1 mmHg, p<0.001). Furthermore, at both age 9 and 15 weeks, FF had a higher heart volume compared to C (9 weeks: 0.31±0.01 vs. 0.28±0.01% bw; 15 weeks: 0.32±0.01 vs. 0.25±0.01% bw, p<0.05) (Fig 3B).

### Enzyme activities: antioxidant enzyme

Three antioxidant enzyme activities were measured: SOD, GPx and CAT (Table 2). Whilst GPx activity remained at the same level for any age (9 and 15 weeks) and tissue (left ventricle and gastrocnemius) studied, SOD and CAT activities varied amongst our groups (FF and C). Fructose supplementation increased SOD levels in the left ventricle and gastrocnemius at age 9 and 15 weeks (9 weeks: heart: 4.68±0.24 vs. 3.21±0.09 USOD/mg protein, p<0.001; gastrocnemius: 4.15±0.18 vs. 2.71±0.14 USOD/mg protein, p<0.001; 15 weeks: heart: 5.35±0.38 vs. 4.03±0.31 USOD/mg protein, p<0.05, gastrocnemius: 5.57±0.29 vs. 4.56±0.36 USOD/mg protein p<0.05). Fructose supplementation also increased CAT activity levels, although this modification only appeared at age 15 weeks in the gastrocnemius (2.01±0.34 vs. 1.01±0.13 nmol H_2_O_2_/min/mg protein, p<0.05). CAT activity levels remained unchanged otherwise.

**Table 2:** Effects of fructose supplementation on GPx, CAT and SOD activities in both left ventricle (LV) and gastrocnemius. (*n = 8* in all conditions).

	*GPx (nmol NADPH/min/mg protein)*		*CAT (nmol H2O2/min/mg protein)*		*SOD (USOD/mg protein)*	
*Age (weeks)*	LV	Gastrocnemius	LV	Gastrocnemius	LV	Gastrocnemius
*9*						
FF	7.72±0.53	1.53±0.35	6.87±0.62	1.933±0.19	4.68±0.24	4.15±0.18
C	7.09±0.19	1.13±0.08	6.51±0.62	1.446±0.22	3.21±0.09***	2.71±0.14***
*15*						
FF	8.25±0.74	1.36±0.12	5.34±0.55	2.009±0.34	5.35±0.38	5.57±0.29
C	7.89±0.40	1.38±0.17	7.83±0.62	1.011±0.13*	4.03±0.31*	4.56±0.36*

FF: fructose supplemented; C: control. Statistical values:

*<0.05,

**<0.01,

***<0.001.

## Discussion

The purpose of this study was to establish a model of type II diabetes and insulin resistance in fructose fed Wistar rats, using a diet as close as possible to human consumption. Indeed Tappy and Lê, (2010)[4] highlighted the lack of studies close to the actual human consumption of fructose: 50g/d for a man, mostly consumed with sweet beverages, beginning as early as 6 years old. Some studies had begun to define more realistic diets using Sprague-Dawley rats [23]. However this specific rat strain is known to be more sensitive towards T2D than Wistar rats [9,10], thus making Sprague-Dawley less representative of the human population, where sensitivity towards T2D varies greatly from an individual to another. Wistar rats are, by their varied sensitivity towards T2D, closer to the human population. The existing studies on Sprague Dawley rats showed that a high fructose diet can induce various signs of metabolic syndrome (hyperglycemia, reduced glucose tolerance, insulin resistance, weight gain…) as well as T2D [5–9]. Our study on Wistar rats using a realistic diet is consistent with these previous findings.

Indeed, 6 weeks of fructose supplementation are sufficient to induce an increase in fasting glycemia (Table 1) associated with a reduced glucose tolerance (shown with the significant differences observed during OGTT, as no baseline exists for this parameter in Wistar rats) (Fig 1A). The same conclusion can be drawn after 12 weeks of fructose supplementation (Table 1, Fig 1B). Regarding fasting glycemia (FF: 98.28±3.21; C: 88.14±2.26 mg/dL), our results are consistent with other studies on control rats (Baker *et al*. [24]: *50-130mg/dL*, Patel *et al*. 2009 [25]: 86.4+/3.6mg/dL, Silva *et al*[26]: 82+/-2mg/dL), but also with studies using a fructose enriched diet (Patel *et al*. 2009 [25], Fructose fed rats: 109.8+/-1.8mg/dL; Silva *et al*[26], Fructose fed rats: 92+/-2mg/dL).

During OGTT, insulin levels were measured concomitantly with glycemia (Fig 1C and 1D). Fructose supplemented rats, both after 6 and 12 weeks of the special diet, showed higher insulin levels when compared to control groups, indicating that 6 weeks of fructose supplementation is sufficient to induce insulin resistance in Wistar rats. This observation is supported by the results of both HOMA 2-IR [12] and ISI-gly [13]. Indeed, HOMA 2-IR, calculated from fasting values of glucose and insulin, provides a better information on hepatic insulin sensitivity [27]. A HOMA 2-IR higher than 1.85 is sufficient to define insulin resistance [28]. After 12 weeks of fructose supplementation FF rats are insulin resistant with an HOMA IR equal to 2.87±0.43. On the other hand ISI-gly is calculated from OGTT data (glucose and insulin levels) and is considered to be a whole body/peripheral insulin sensitivity index [29]. Isi-gly, unlike HOMA 2-IR does not possess defined cut-off values. However, a lower ISI-gly value indicates reduced insulin sensitivity. In our study ISI-gly is lowered as early as after 6 weeks of fructose supplementation compared to the control diet (FF: 0.21±0.02 vs. C: 0.37±0.10, p<0.05) but also after 12 weeks of fructose supplementation (FF: 0.076±0.0069 vs. C: 0.113±0.009, p<0.01). These two markers indicate that after 6 weeks of fructose supplementation rats were only less sensitive towards insulin, but after 12 weeks of fructose supplementation rats were insulin resistant (Fig 2A and 2B).

In addition to the T2D signs, fructose supplementation also induced some metabolic syndrome markers. Indeed, in our study fructose supplementation generates weight gain (Table 1) but only after 12 weeks of fructose enriched diet. No effect of fructose supplementation has been observed on weight gain at age 9 weeks. Interestingly, the increased energy intake observed in FF rats was mainly due to fructose supplementation and not to food intake (S1 Fig). Although this was observed during the entire protocol period, weight gain was only reported at the end of this time.

Metabolic syndrome signs include dyslipidemia. While well documented in Sprague Dawley rats, dyslipidemia does not appear to be constantly induced by fructose supplementation in Wistar rats. Indeed, studies vary regarding variations in lipid levels. Some indicated a higher cholesterol and an unchanged triglycerides level [30], while others showed the contrary [9][31], and some observed no variation in lipid levels [32]. In our study, neither triglycerides nor total cholesterol have been significantly modified (Table 1). In our study, the NEFA level has only a trend to increase in fructose supplemented, rats (p = 0.054). This observation is not in concordance with the general knowledge about NEFA. Elevated plasma NEFA are also correlated with numerous metabolic and cardiovascular markers such as impaired endothelial function, higher blood pressure [33], as well as T2D in humans [34]. NEFA bound to plasma albumin and is transported mainly to the liver, where they are the main substrate for triglyceride synthesis [33]. From lipid levels, we measured different ratios, only the Total Cholesterol/Triglycerides ratio, increased at age 9 weeks with fructose supplementation. The total Cholesterol/Triglycerides ratio can be used to predict the presence of small dense LDL: the higher the ratio, the higher the presence of small dense LDL [16] and thus the higher the risk of cardiovascular disease [35]. Surprisingly, it did not remain the same later (age 15 weeks). This can be explained by the fact that at age 9 weeks Triglycerides seemed to be higher under fructose supplementation while Total Cholesterol levels remained unchanged. Opposite variations seemed to be observed at age 15 weeks. The other two measured ratios (NEFA/Albumin, NEFA/cholesterol) remained unchanged in all conditions (Table 1). Our result is surprising as fructose is already known to induce dyslipidemia both in human and Sprague Dawley rats [4], [5]. However, Wistar rats are less sensitive towards T2D than Sprague-Dawley rats [9,10] and may need a longer duration and/or higher concentrated fructose supplementation to develop dislypidemia. Indeed, in Silva *et al*. study [26], 10% of fructose in the drink for 18 weeks induced dislipidemia, just as 35% for 30 days in Thornburn *et al*. study [36].

Systolic blood pressure is also higher in the early days of fructose supplementation (Fig 3), another symptom of metabolic syndrome. Fructose supplementation also increased heart volume for the entire duration of the study. Although blood pressure has not been measured at the end of the study, higher heart volume observed at this time can result from higher blood pressure [37]; [38]. Thus it may be possible to suggest that systolic blood pressure is also increased at the end of 12 weeks of fructose supplementation. However, further studies are needed to confirm this hypothesis. Renal function was studied to determine whether the higher blood pressure was related to renal failure. For this purpose theoretical creatinine clearance was calculated using the Cockcroft and Gault formula, as it has already been used in rats [18]. However, unlike for Munich-Wistar rats, there is no baseline data for Wistar rats, values vary from one study to another: from 0.2mL/min [18] to 3.25mL/min [39]. Thus, we only compared our control group to our Fructose enriched group. As fructose supplemented rats have a higher (9 weeks old) or an equal (15 weeks old) theoretical creatinine clearance, the systolic blood pressure rise observed at age 8 weeks does not seem to be related to renal failure. According to Elliott et al [5], fructose enriched diet increased blood pressure, however its mechanism remains unknown.

As for antioxidant enzyme activities, all the measured enzymes have a different pattern regarding fructose supplementation. GPx activity remained unchanged for the duration of the study in every studied tissue. SOD was increased both in the left ventricle and gastrocnemius at all ages. CAT activity level was increased only in the gastrocnemius at age 15 weeks. T2D is already known to negatively impact the antioxidant system inducing harmful consequences (such as lipid peroxydation or DNA mutation) [40]; [41]. It has been shown that T2D induces increased reactive oxygen species (ROS) levels. However, the antioxidant enzyme activities vary from one study to another: some showed a decrease in antioxidant enzyme activities levels [42]; [43], while others have shown an increase [44]; [45]. In our study, the progressive development of T2D induced the progressive increase of the antioxidant enzyme activities (SOD then CAT).

## Conclusion

Fructose supplementation (20–25% w/v for 12 weeks) in Wistar rats induced progressive development of some metabolic syndrome markers (hypertension and increased body weight) as well as an insulin resistance. Thus our 12 week diet enabled us to study the first few steps leading to T2D. Longer diet duration should lead to T2D. The originality of our model is the use of a fructose-enriched diet close to the actual consumption of young children. Further studies in this animal model should help to better understand metabolic syndrome and type 2 diabetes consequences and their prevention.

## Supporting Information

S1 Fig Effects of fructose supplementation on energy (A) and food intake (B). FF: fructose supplemented; C: control. From age 4 to 9 weeks n = 16 rats for both groups, from age 9 to 15 weeks n = 8 rats for both groups. Statistical values: * p<0.05, ** p<0.01, ***p<0.001.
(TIF)

S1 Table Raw data for fasting glycaemia, heartweight, systolic blood pressure and antioxidant enzyme activities. NM: Non Measured, systolic blood pressure was only measured once during the study. Systolic blood pressure: 5 measures were realized consecutively, results shown are the mean of those 5 consecutive measures. ND: Non Determined: for fasting glycemia, the animal was too stressed to allow us to do the measurement, for heartweight, heart was cut and frozen in liquid nitrogen before the measurement, values were thus unreliable.
(TIF)

S2 Table Raw data for bodyweight. ND: Non determined.
(TIF)

S3 Table Raw data for food intake. ND: Non determined.
(TIF)

S4 Table Raw data for energy intake. ND: Non determined.
(TIF)

S5 Table Raw data for glycemia during OGTT at both 9 and 13 weeks. ND: Non determined. Animal was to stressed during the manipulation, no blood was recolted.
(TIF)

S6 Table Raw data for insulin levels during OGTT at both 9 and 13 weeks. ND: Non determined. Plasma supplies were not sufficient for each time point to enable us to do the measure in triplicate as require for the ELISA-assay.
(TIF)

S7 Table Raw data for biochemical analyses. ND: Non determined. Plasma supplies were not sufficient to enable the analysis.
(TIF)

## References

[1] Whiting DR, Guariguata L, Weil C, Shaw J. IDF Diabetes Atlas: Global estimates of the prevalence of diabetes for 2011 and 2030. Diabetes Res Clin Pract. 2011;94: 311–321. 10.1016/j.diabres.2011.10.029 22079683

[2] Wild S, Roglic G, Green A, Sicree R, King H. Global Prevalence of Diabetes Estimates for the year 2000 and projections for 2030. Diabetes Care. 2004;27: 1047–1053. 10.2337/diacare.27.5.1047 15111519

[3] Malik VS, Popkin BM, Bray GA, Després J-P, Willett WC, Hu FB. Sugar-Sweetened Beverages and Risk of Metabolic Syndrome and Type 2 Diabetes A meta-analysis. Diabetes Care. 2010;33: 2477–2483. 10.2337/dc10-1079 20693348 PMC2963518

[4] Tappy L, Lê K-A. Metabolic Effects of Fructose and the Worldwide Increase in Obesity. Physiol Rev. 2010;90: 23–46. 10.1152/physrev.00019.2009 20086073

[5] Elliott SS, Keim NL, Stern JS, Teff K, Havel PJ. Fructose, weight gain, and the insulin resistance syndrome. Am J Clin Nutr. 2002;76: 911–922. 12399260 10.1093/ajcn/76.5.911

[6] Havel PJ. Dietary Fructose: Implications for Dysregulation of Energy Homeostasis and Lipid/Carbohydrate Metabolism. Nutr Rev. 2005;63: 133–157. 10.1111/j.1753-4887.2005.tb00132.x 15971409

[7] Huang B-W, Chiang M-T, Yao H-T, Chiang W. The effect of high-fat and high-fructose diets on glucose tolerance and plasma lipid and leptin levels in rats. Diabetes Obes Metab. 2004;6: 120–126. 14746577 10.1111/j.1462-8902.2004.00323.x

[8] Hwang I-S, Ho H, Hoffman BB, Reaven GM. Fructose-induced insulin resistance and hypertension in rats. Hypertension. 1987;10: 512–516. 3311990 10.1161/01.hyp.10.5.512

[9] de Moura RF, Ribeiro C, de Oliveira JA, Stevanato E, de Mello MAR. Metabolic syndrome signs in Wistar rats submitted to different high-fructose ingestion protocols. Br J Nutr. 2008;101: 1178 10.1017/S0007114508066774 19007450

[10] Ribeiro RT, Lautt WW, Legare DJ, Macedo MP. Insulin resistance induced by sucrose feeding in rats is due to an impairment of the hepatic parasympathetic nerves. Diabetologia. 2005;48: 976–983. 10.1007/s00125-005-1714-6 15830187 PMC2925889

[11] Bettaieb A, Vazquez Prieto MA, Rodriguez Lanzi C, Miatello RM, Haj FG, Fraga CG, et al (-)-Epicatechin mitigates high-fructose-associated insulin resistance by modulating redox signaling and endoplasmic reticulum stress. Free Radic Biol Med. 2014;72: 247–256. 10.1016/j.freeradbiomed.2014.04.011 24746618 PMC4077617

[12] Matthews DR, Hosker JP, Rudenski AS, Naylor BA, Treacher DF, Turner RC. Homeostasis model assessment: insulin resistance and beta-cell function from fasting plasma glucose and insulin concentrations in man. Diabetologia. 1985;28: 412–419. 3899825 10.1007/BF00280883

[13] Belfiore F, Iannello S, Volpicelli G. Insulin Sensitivity Indices Calculated from Basal and OGTT-Induced Insulin, Glucose, and FFA Levels. Mol Genet Metab. 1998;63: 134–141. 10.1006/mgme.1997.2658 9562967

[14] Ndlovu T, Chimonyo M, Okoh AI, Muchenje V, Dzama K, Raats JG. Assessing the nutritional status of beef cattle: current practices and future prospects. Afr J Biotechnol. 2007;6 Available: http://www.ajol.info/index.php/ajb/article/download/58187/46550

[15] Pickart L. Increased ratio of plasma free fatty acids to albumin during normal aging and in patients with coronary heart disease. Atherosclerosis. 1983;46: 21–28. 10.1016/0021-9150(83)90160-0 6838692

[16] Yoshida A, Kouwaki M, Matsutani Y, Fukuchi Y, Naito M. Usefulness of serum total cholesterol/triglyceride ratio for predicting the presence of small, dense LDL. J Atheroscler Thromb. 2004;11: 215–219. 15356381 10.5551/jat.11.215

[17] Cockcroft DW, Gault MH. Prediction of creatinine clearance from serum creatinine. Nephron. 1976;16: 31–41. 1244564 10.1159/000180580

[18] Yuan G, Deng J, Wang T, Zhao C, Xu X, Wang P, et al Tissue Kallikrein Reverses Insulin Resistance and Attenuates Nephropathy in Diabetic Rats by Activation of PI3 kinase/Akt and AMPK Signaling Pathways. Endocrinology. 2007;148: 2016–2026. 10.1210/en.2006-0602 17272402 PMC2084357

[19] Misra HP, Fridovich I. The role of superoxide anion in the autoxidation of epinephrine and a simple assay for superoxide dismutase. J Biol Chem. 1972;247: 3170–3175. 4623845

[20] Amérand A, Vettier A, Sébert P, Moisan C. Does hydrostatic pressure have an effect on reactive oxygen species in the eel? Undersea Hyperb Med J Undersea Hyperb Med Soc Inc. 2006;33: 157–160. 16869528

[21] Beers RF, Sizer IW. A spectrophotometric method for measuring the breakdown of hydrogen peroxide by catalase. J Biol Chem. 1952;195: 133–140. 14938361

[22] Ross SW, Dalton DA, Kramer S, Christensen BL. Physiological (antioxidant) responses of estuarine fishes to variability in dissolved oxygen. Comp Biochem Physiol Toxicol Pharmacol CBP. 2001;130: 289–303. 10.1016/s1532-0456(01)00243-5 11701386

[23] Pranprawit A, Wolber FM, Heyes JA, Molan AL, Kruger MC. Short-term and long-term effects of excessive consumption of saturated fats and/or sucrose on metabolic variables in Sprague Dawley rats: a pilot study. J Sci Food Agric. 2013;93: 3191–3197. 10.1002/jsfa.6240 23712415

[24] Baker HJ, Lindsey JR, Weisbroth SH, editors. Biology and diseases. New York, NY: Acad. Press; 1979.

[25] Patel J, Iyer A, Brown L. Evaluation of the chronic complications of diabetes in a high fructose diet in rats. Indian J Biochem Biophys. 2009;46: 66–72. 19374256

[26] Silva RJ da, Bernardes N, Brito J de O, Sanches IC, Irigoyen MC, Angelis KD. Simvastatin-induced cardiac autonomic control improvement in fructose-fed female rats. Clinics. 2011;66: 1793–1796. 10.1590/S1807-59322011001000019 22012053 PMC3180142

[27] Carnevale Schianca GP, Sainaghi PP, Castello L, Rapetti R, Limoncini AM, Bartoli E. Comparison between HOMA-IR and ISI-gly in detecting subjects with the metabolic syndrome. Diabetes Metab Res Rev. 2006;22: 111–117. 10.1002/dmrr.560 16052601

[28] Gayoso-Diz P, Otero-González A, Rodriguez-Alvarez MX, Gude F, García F, Francisco AD, et al Insulin resistance (HOMA-IR) cut-off values and the metabolic syndrome in a general adult population: effect of gender and age: EPIRCE cross-sectional study. BMC Endocr Disord. 2013;13: 47 10.1186/1472-6823-13-47 24131857 PMC4016563

[29] Matsuda M, DeFronzo RA. Insulin sensitivity indices obtained from oral glucose tolerance testing: comparison with the euglycemic insulin clamp. Diabetes Care. 1999;22: 1462–1470. 10480510 10.2337/diacare.22.9.1462

[30] Dimo T, Rakotonirina SV, Tan PV, Azay J, Dongo E, Cros G. Leaf methanol extract of Bidens pilosa prevents and attenuates the hypertension induced by high-fructose diet in Wistar rats. J Ethnopharmacol. 2002;83: 183–191. 10.1016/S0378-8741(02)00162-9 12426085

[31] Zamami Y, Takatori S, Goda M, Koyama T, Iwatani Y, Jin X, et al Royal Jelly Ameliorates Insulin Resistance in Fructose-Drinking Rats. Biol Pharm Bull. 2008;31: 2103–2107. 18981581 10.1248/bpb.31.2103

[32] Haeri MR, Izaddoost M, Ardekani MRS, Nobar MR, White KN. The effect of fenugreek 4-hydroxyisoleucine on liver function biomarkers and glucose in diabetic and fructose-fed rats. Phytother Res. 2009;23: 61–64. 10.1002/ptr.2557 18680121

[33] Karpe F, Dickmann JR, Frayn KN. Fatty Acids, Obesity, and Insulin Resistance: Time for a Reevaluation. Diabetes. 2011;60: 2441–2449. 10.2337/db11-0425 21948998 PMC3178283

[34] Kahn SE, Hull RL, Utzschneider KM. Mechanisms linking obesity to insulin resistance and type 2 diabetes. Nature. 2006;444: 840–846. 10.1038/nature05482 17167471

[35] Lamarche B, Lemieux I, Després JP. The small, dense LDL phenotype and the risk of coronary heart disease: epidemiology, patho-physiology and therapeutic aspects. Diabetes Metab. 1999;25: 199–211. 10499189

[36] Thorburn AW, Storlien LH, Jenkins AB, Khouri S, Kraegen EW. Fructose-induced in vivo insulin resistance and elevated plasma triglyceride levels in rats. Am J Clin Nutr. 1989;49: 1155–1163. 2658534 10.1093/ajcn/49.6.1155

[37] Sen S, Tarazi RC, Khairallah PA, Bumpus FM. Cardiac hypertrophy in spontaneously hypertensive rats. Circ Res. 1974;35: 775–781. 4371062 10.1161/01.res.35.5.775

[38] Doggrell SA, Brown L. Rat models of hypertension, cardiac hypertrophy and failure. Cardiovasc Res. 1998;39: 89–105. 10.1016/S0008-6363(98)00076-5 9764192

[39] Egbuonu ACC, Ezeanyika LUS. L-arginine Exposure Improves Renal Function Markers of Metabolic Syndrome in Female Rats. Am J Biochem Mol Biol. 2013;3: 50–60. 10.3923/ajbmb.2013.50.60

[40] Susztak K, Raff AC, Schiffer M, Böttinger EP. Glucose-Induced Reactive Oxygen Species Cause Apoptosis of Podocytes and Podocyte Depletion at the Onset of Diabetic Nephropathy. Diabetes. 2006;55: 225–233. 10.2337/diabetes.55.01.06.db05-0894 16380497

[41] Wright E, Scism-Bacon J, Glass L. Oxidative stress in type 2 diabetes: the role of fasting and postprandial glycaemia. Int J Clin Pract. 2006;60: 308–314. 10.1111/j.1368-5031.2006.00825.x 16494646 PMC1448694

[42] Bhatia S, Shukla R, Venkata Madhu S, Kaur Gambhir J, Madhava Prabhu K. Antioxidant status, lipid peroxidation and nitric oxide end products in patients of type 2 diabetes mellitus with nephropathy. Clin Biochem. 2003;36: 557–562. 10.1016/S0009-9120(03)00094-8 14563450

[43] Rector RS, Uptergrove GM, Borengasser SJ, Mikus CR, Morris EM, Naples SP, et al Changes in skeletal muscle mitochondria in response to the development of type 2 diabetes or prevention by daily wheel running in hyperphagic OLETF rats. Am J Physiol—Endocrinol Metab. 2010;298: E1179–E1187. 10.1152/ajpendo.00703.2009 20233940 PMC2886529

[44] Aydın A, Orhan H, Sayal A, Özata M, Şahin G, Işımer A. Oxidative stress and nitric oxide related parameters in type II diabetes mellitus: effects of glycemic control. Clin Biochem. 2001;34: 65–70. 10.1016/S0009-9120(00)00199-5 11239518

[45] Seghrouchni I, Drai J, Bannier E, Rivière J, Calmard P, Garcia I, et al Oxidative stress parameters in type I, type II and insulin-treated type 2 diabetes mellitus; insulin treatment efficiency. Clin Chim Acta. 2002;321: 89–96. 10.1016/S0009-8981(02)00099-2 12031597

