# Supplementary figures and images for: Progressive Induction of Type 2 Diabetes: Effects of a Reality–Like Fructose Enriched Diet in Young Wistar Rats
Source: PLoS One. 2016 Jan 22;11(1):e0146821. doi: 10.1371/journal.pone.0146821 (PMC4723014; doi:10.1371/journal.pone.0146821)

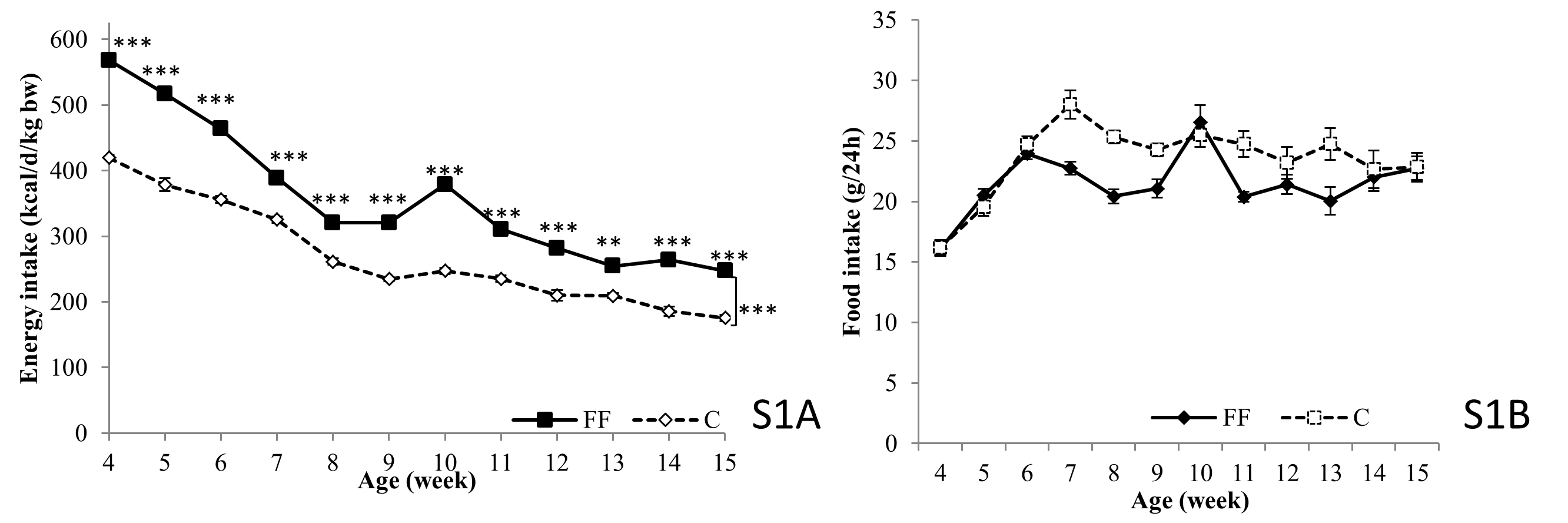

Supplement: S1 Fig — FF: fructose supplemented; C: control. From age 4 to 9 weeks n = 16 rats for both groups, from age 9 to 15 weeks n = 8 rats for both groups. Statistical values: * p<0.05, ** p<0.01, ***p<0.001. (TIF) [file pone.0146821.s001.tif]

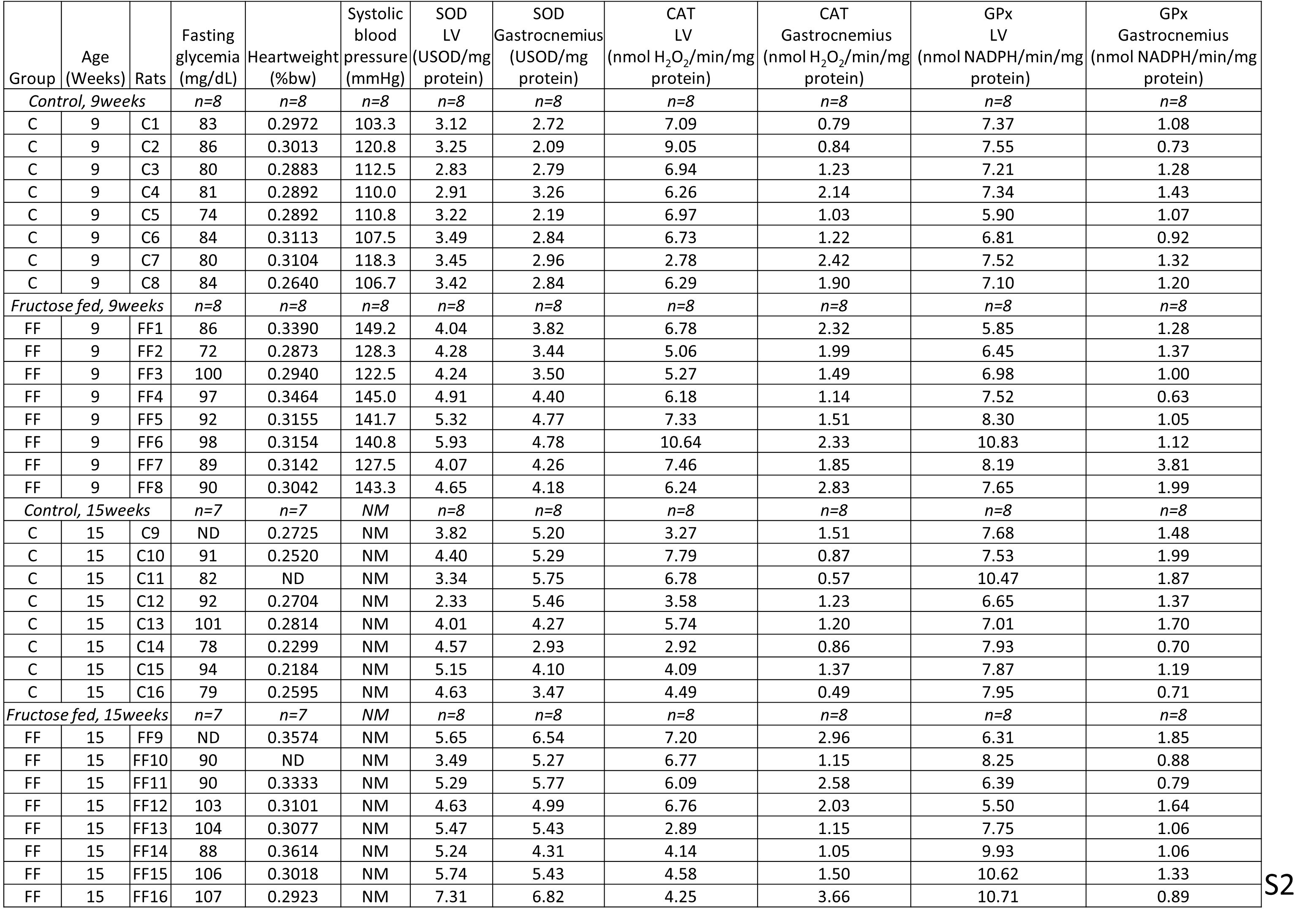

Supplement: S1 Table — NM: Non Measured, systolic blood pressure was only measured once during the study. Systolic blood pressure: 5 measures were realized consecutively, results shown are the mean of those 5 consecutive measures. ND: Non Determined: for fasting glycemia, the animal was too stressed to allow us to do the measurement, for heartweight, heart was cut and frozen in liquid nitrogen before the measurement, values were thus unreliable. (TIF) [file pone.0146821.s002.TIF]

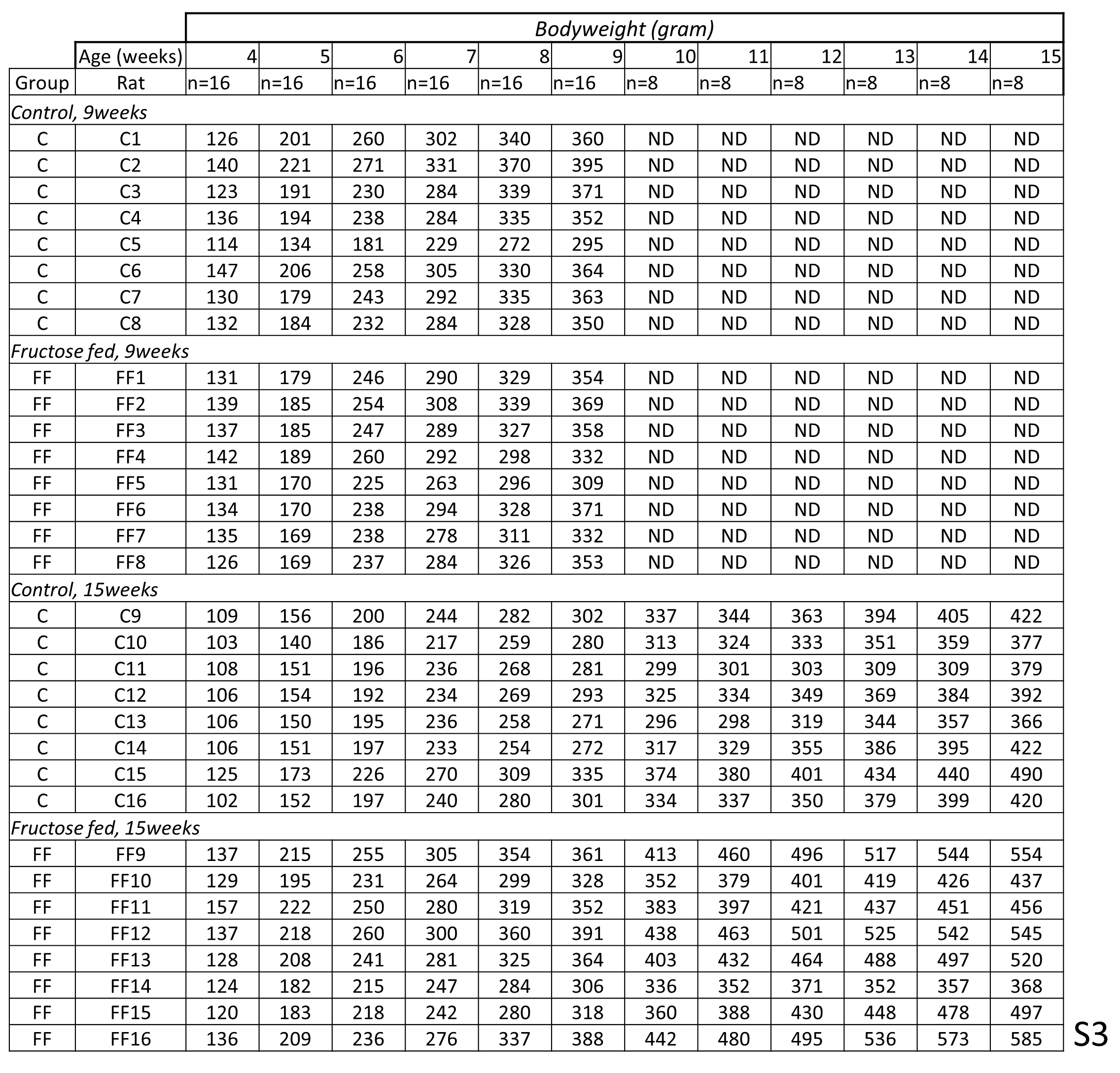

Supplement: S2 Table — ND: Non determined. (TIF) [file pone.0146821.s003.TIF]

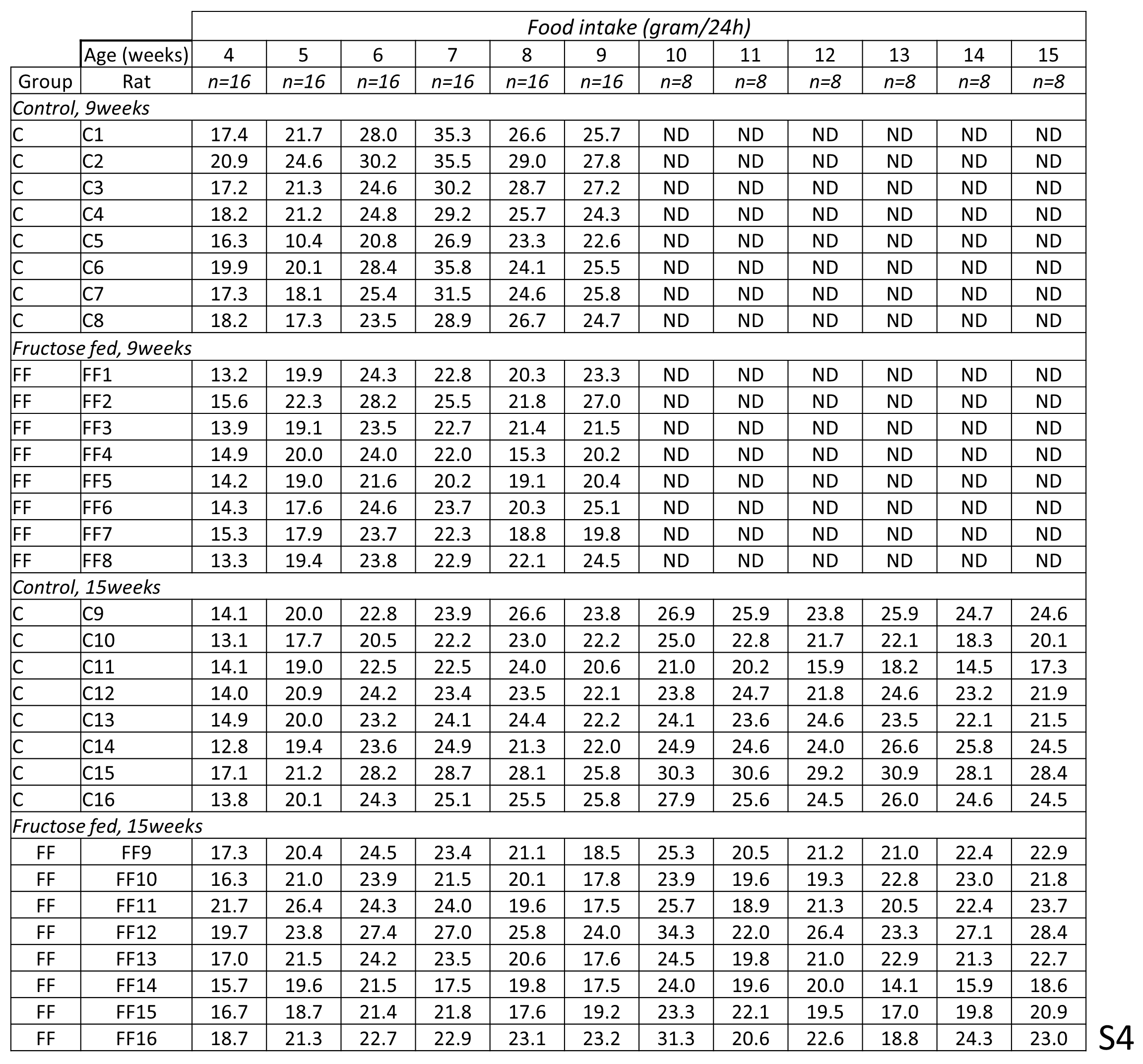

Supplement: S3 Table — ND: Non determined. (TIF) [file pone.0146821.s004.tif]

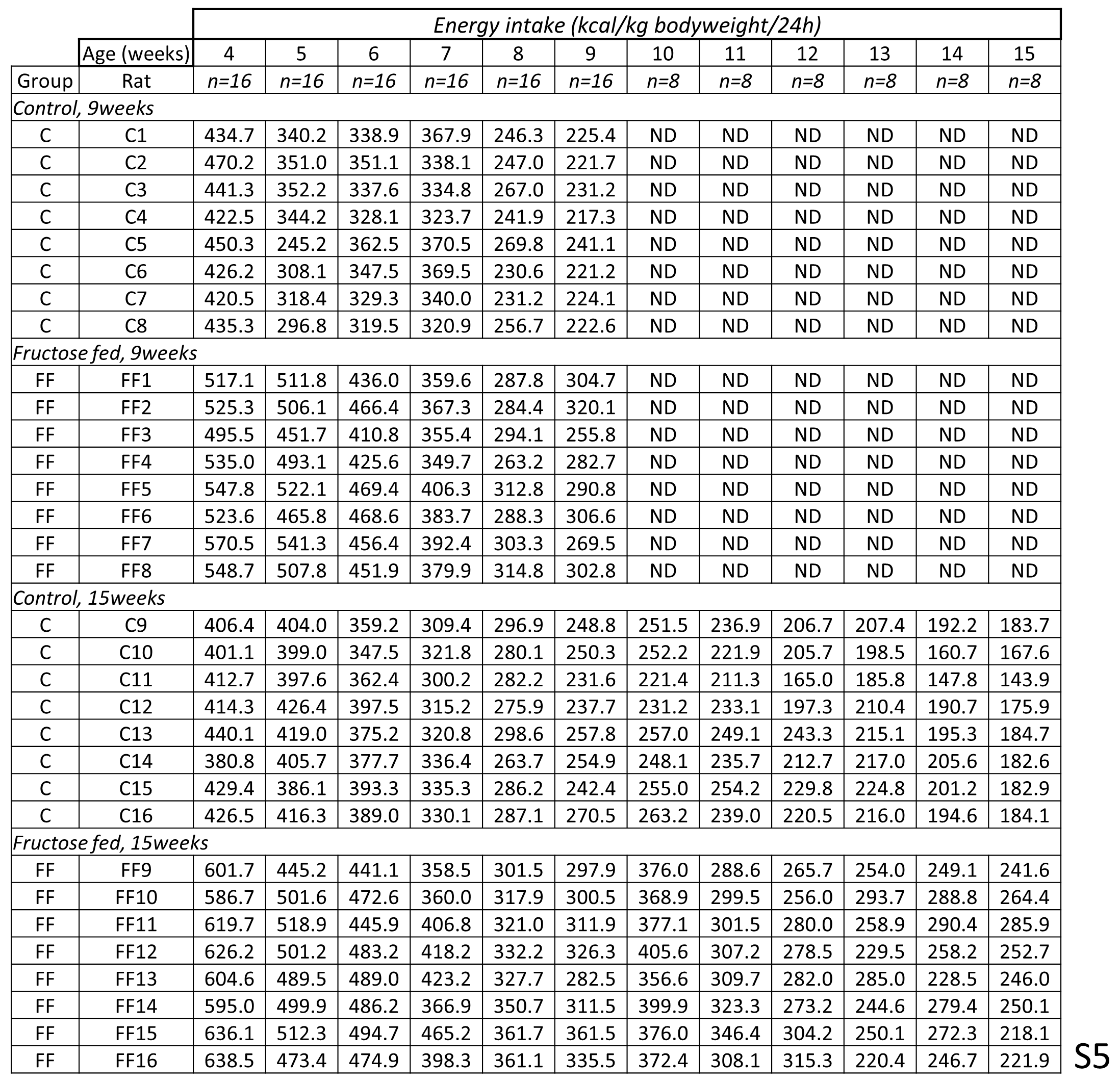

Supplement: S4 Table — ND: Non determined. (TIF) [file pone.0146821.s005.TIF]

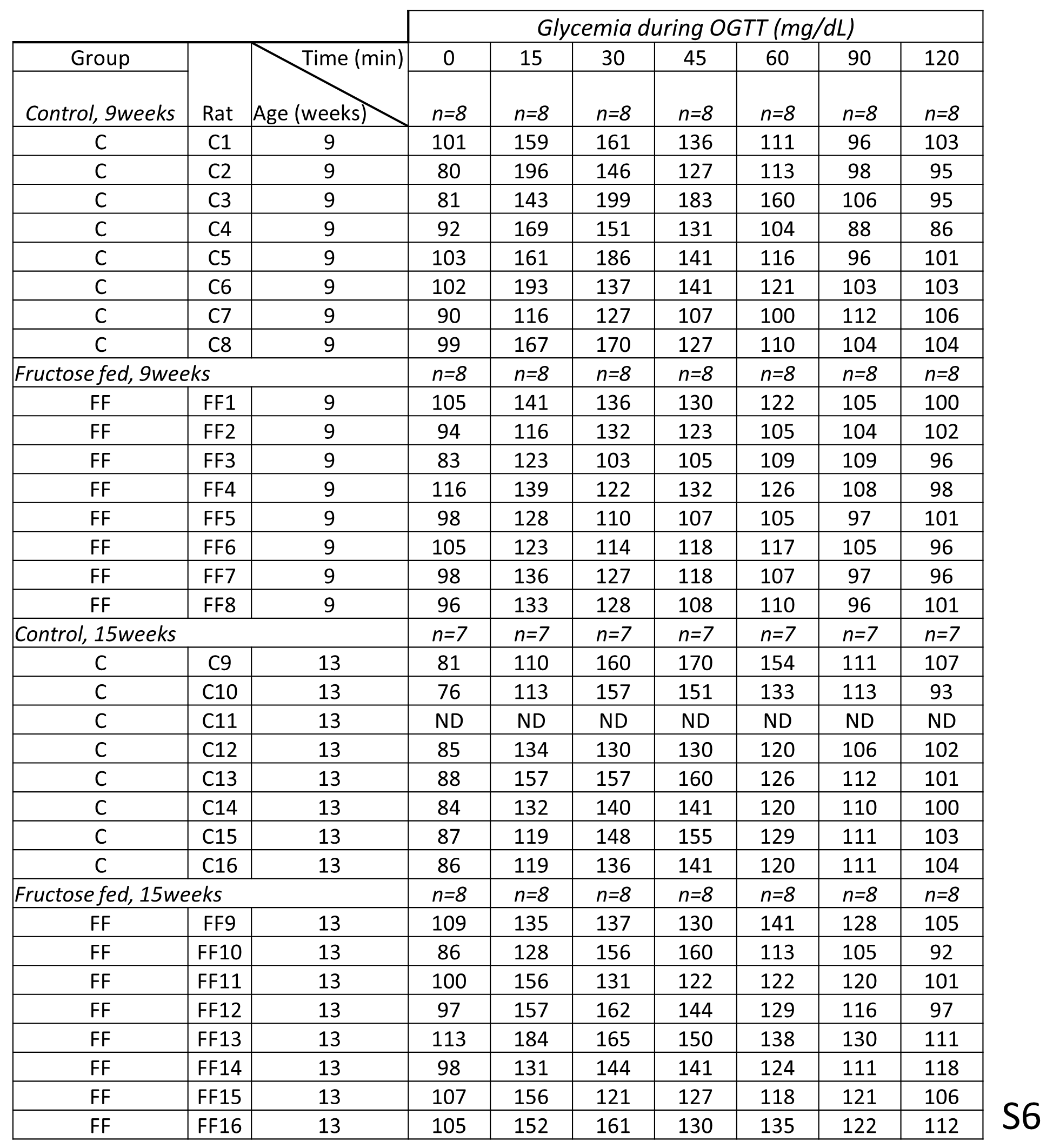

Supplement: S5 Table — ND: Non determined. Animal was to stressed during the manipulation, no blood was recolted. (TIF) [file pone.0146821.s006.TIF]

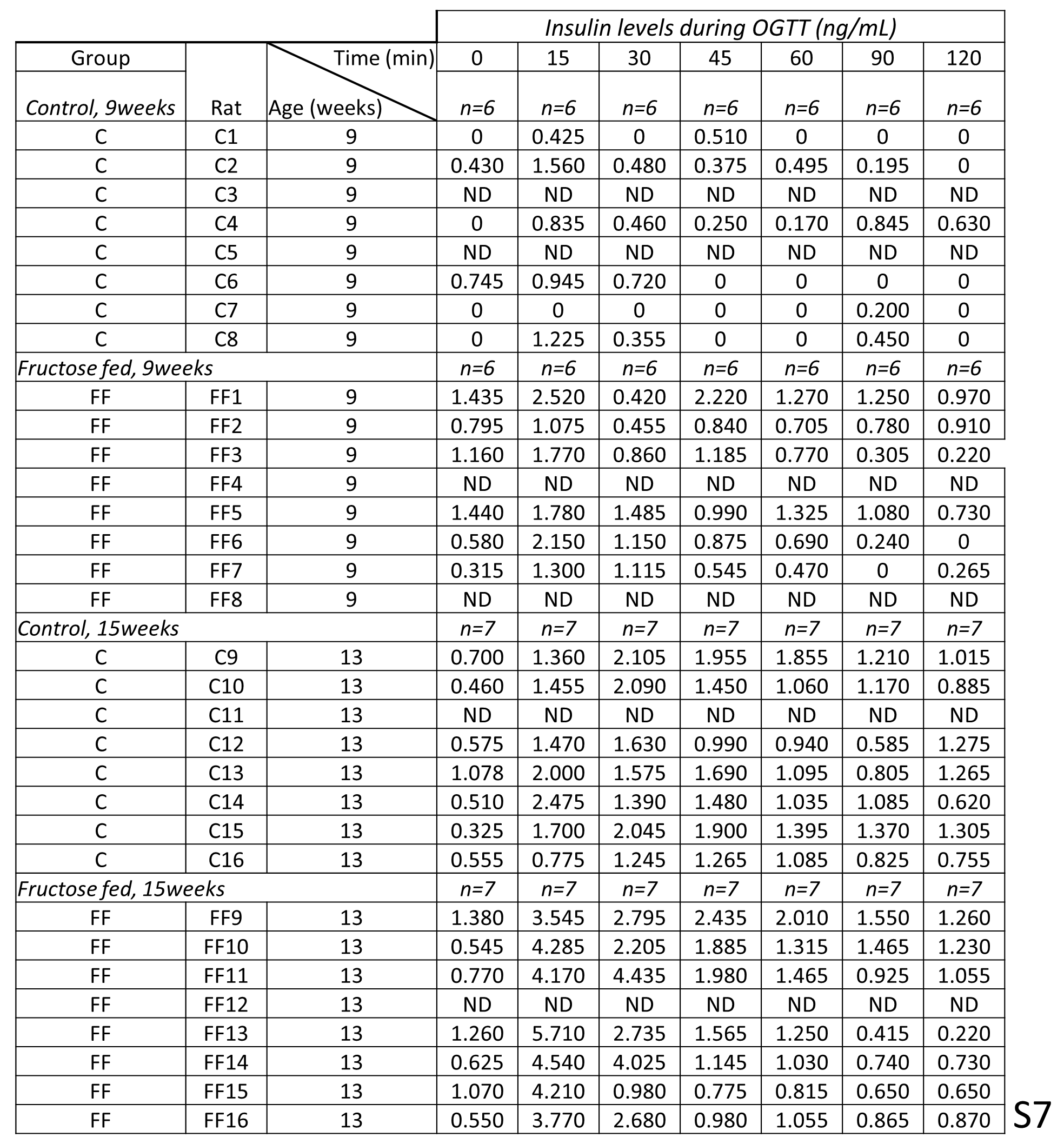

Supplement: S6 Table — ND: Non determined. Plasma supplies were not sufficient for each time point to enable us to do the measure in triplicate as require for the ELISA-assay. (TIF) [file pone.0146821.s007.TIF]

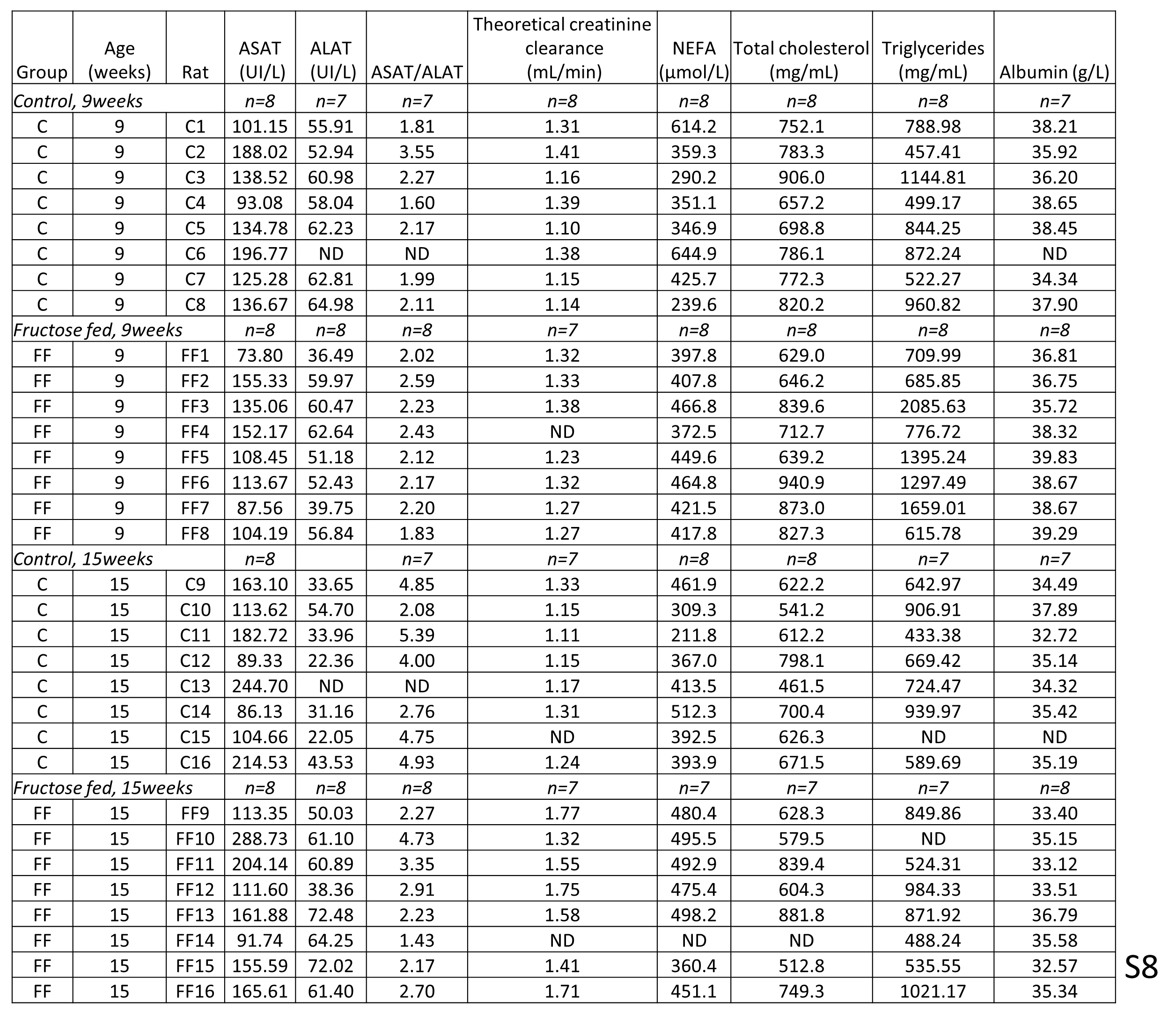

Supplement: S7 Table — ND: Non determined. Plasma supplies were not sufficient to enable the analysis. (TIF) [file pone.0146821.s008.TIF]
